# Patient‐reported tolerability of veliparib combined with cisplatin and etoposide for treatment of extensive stage small cell lung cancer: Neurotoxicity and adherence data from the ECOG ACRIN cancer research group E2511 phase II randomized trial

**DOI:** 10.1002/cam4.3416

**Published:** 2020-08-28

**Authors:** Laurie E. Steffen McLouth, Fengmin Zhao, Taofeek K. Owonikoko, Josephine L. Feliciano, Nisha A. Mohindra, Suzanne E. Dahlberg, James L. Wade, Gordan Srkalovic, Bradley W. Lash, Joseph W. Leach, Ticiana A. Leal, Charu Aggarwal, David Cella, Suresh S. Ramalingam, Lynne I. Wagner

**Affiliations:** ^1^ Department of Behavioral Science Center for Health Equity Transformation University of Kentucky College of Medicine Lexington KY USA; ^2^ Dana‐Farber Cancer Institute & ECOG‐ACRIN Biostatistics Center Boston MA USA; ^3^ Emory University Atlanta GA USA; ^4^ Johns Hopkins University Baltimore MD USA; ^5^ Northwestern University Chicago IL USA; ^6^ Heartland NCORP Decatur IL USA; ^7^ Sparrow Herbert‐Herman Cancer Center Lansing MI USA; ^8^ Guthrie Clinic – Robert Packer Hospital Sayre USA; ^9^ Metro Minnesota NCORP Minneapolis MN USA; ^10^ University of Wisconsin Carbone Cancer Center Madison WI USA; ^11^ University of Pennsylvania Philadelphia PA USA; ^12^ Department of Social Sciences & Health Policy Wake Forest School of Medicine Winston‐Salem NC USA

**Keywords:** chemotherapy‐induced peripheral neuropathy, patient‐reported outcomes, small cell, tolerability, veliparib

## Abstract

**Objectives:**

The ECOG‐ACRIN Cancer Research Group trial E2511 recently demonstrated a potential benefit for the addition of veliparib to cisplatin‐etoposide (CE) in patients with extensive stage small cell lung cancer (ES‐SCLC) in a phase II randomized controlled trial. Secondary trial endpoints included comparison of the incidence and severity of neurotoxicity, hypothesized to be lower in the veliparib arm, and tolerability of the addition of veliparib to CE. Physician‐rated and patient‐reported neurotoxicity was also compared.

**Materials and Methods:**

Patients randomized to veliparib plus CE (n = 64) or placebo plus CE (n = 64) completed the 11‐item Functional Assessment of Cancer Therapy Gynecologic Oncology Group Neurotoxicity (questionnaire pre‐treatment, end of cycle 4 [ie 3 months after randomization] and 3 months post‐treatment [ie 6‐months]). Adherence analysis was based on treatment forms.

**Results and Conclusion:**

No significant differences in mean or magnitude of change in neurotoxicity scores were observed between treatment arms at any time point. However, patients in the placebo arm reported worsening neurotoxicity from baseline to 3‐months (M difference = −1.5, *P* = .045), compared to stable neurotoxicity in the veliparib arm (M difference = −0.2, *P* = .778). Weakness was the most common treatment‐emergent (>50%) and moderate to severe (>16%) symptom reported, but did not differ between treatment arms. The proportion of adherence to oral therapy in the overall sample was 75%. Three percent of patients reported clinically significant neurotoxicity that was not captured by physician assessment. Neurotoxicity scores were not different between treatment arms. The addition of veliparib to CE appeared tolerable, though weakness should be monitored.

**ClinicalTrials.gov Identifier:**

NCT01642251.

## Graphical abstract

We examined patient‐reported tolerability of the addition of a PARP inhibitor, veliparib, to cisplatin‐etoposide for treatment of extensive stage small cell lung cancer in randomized controlled trial E2511. The addition of veliparib to cisplatin‐etoposide was tolerable according to a patient‐reported outcome and adherence. Future studies should assess whether veliparib may have a protective effect against chemotherapy‐induced peripheral neuropathy.

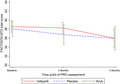

## 1 INTRODUCTION

Cisplatin‐etoposide is a common first line treatment for extensive stage small cell lung cancer (ES‐SCLC).^1^ Although ES‐SCLC initially responds well to this regimen, treatment resistance inevitably occurs,^2^ necessitating novel therapeutic agents. The impact of novel agents and combinations of targeted agents on toxicities and tolerability of standard therapy is relatively unknown. Concurrent, comprehensive assessment of treatment tolerability from the patient's perspective is needed to ensure patients receive maximal benefit from new therapies without jeopardizing quality of life.

The ECOG‐ACRIN Cancer Research Group recently reported a potential signal for the addition of veliparib, a poly (ADP) ribose polymerase (PARP) inhibitor, to cisplatin and etoposide (CE) to improve progression free survival among a subset of treatment‐naïve patients with ES‐SCLC in a double‐blinded randomized controlled trial (E2511).^3^ The treatment arms appeared to have comparable toxicity based on CTCAE version 4.0 ratings, with the exception of higher hematologic toxicities with the addition of veliparib. Specifically, grade 3 or higher lymphopenia and neutropenia occurred more often in the veliparib arm, though did not disrupt treatment. No instances of grade 3 or higher neuropathy, one of the main toxicities associated with platinum‐based therapies, were reported in either treatment arm.

Although the trial's results suggest the addition of veliparib to CE is tolerable, CTCAE grades may underestimate the impact of treatment on patients,^4^ underscoring the need for complementary patient‐reported outcomes to assess tolerability. Psychometrically validated patient‐reported outcomes (PRO) provide robust information about the severity, frequency, and/or functional impact of a symptom associated with treatment.^5^, ^6^ This information is critical, as the extent to which a patient is willing to tolerate a symptom for a potential anti‐tumor benefit depends in part on the subjective experience of a symptom.

The current report provides in‐depth analysis of treatment toxicity and tolerability among patients randomized to either cisplatin‐etoposide + veliparib (CE + V) or cisplatin‐etoposide + placebo (CE + P) in E2511. Patient‐reported treatment toxicity focused on neurotoxicity, a common,^7^ disruptive,^8^, ^9^ and largely refractory toxicity^10^ for many patients treated with CE that can worsen even after cisplatin is discontinued.^11^, ^12^ The patient‐reported outcomes component of this trial was designed to test the hypothesis that patients in the veliparib arm would experience less neurotoxicity than patients in the placebo arm. This hypothesis was based on preclinical data indicating a potential protective role for PARP enzyme inhibition in experimental models of diabetic neuropathy, suggesting veliparib may have a neuroprotective effect.^13^, ^14^ Secondary objectives were to identify treatment‐emergent and the most prevalent moderate to severe symptoms of neurotoxicity, compare adherence to oral therapy (ie veliparib vs placebo) as a behavioral indicator of treatment tolerability, and compare CTCAE rated neuropathy with patient‐reported neurotoxicity. Together, PRO data are intended to provide a more comprehensive assessment of this regimen's tolerability and inform future clinical trial design through generating precise and robust measures of key domains.

## 2 MATERIALS AND METHODS

E2511 design and primary trial results have been reported.^3^ E2511 was a phase I/II double‐blinded randomized controlled trial comparing progression free survival in treatment‐naive ES‐SCLC patients receiving frontline (CE + V) to (CE + P). Secondary objectives included a comparison of chemotherapy‐induced peripheral neuropathy (CIPN) between treatment arms. The protocol was approved by the Institutional Review Boards at each registering institution. Written informed consent was obtained from all patients. The study was activated on 24 October 2013 and terminated on 2 July 2015. ClinicalTrials.gov Identifier: NCT01642251.

### 2.1 Participants

The full eligibility criteria for this trial have been reported.^3^ Patients were eligible if they were at least 18 years old with untreated, newly diagnosed, and pathologically confirmed ES‐SCLC, ECOG performance status of 0 or 1, and measurable disease per RECIST 1.1.

### 2.2 Data collection

Patient‐reported outcome assessments were administered on paper pre‐randomization (ie baseline), at completion of cycle 4 (ie 3 months), and 3 months after completion of cycle 4 (ie 6 months).

### 2.3 Measures

#### 2.3.1 Patient‐reported Neurotoxicity

The English version of the 11‐item Functional Assessment of Cancer Therapy/Gynecologic Oncology Group‐Neurotoxicity Additional Concerns (FACT/GOG‐Ntx Version 4)^15^ was administered via paper to assess patient‐reported neurotoxicity. The FACT/GOG‐Ntx asks patients to rate neurotoxicity symptoms (eg “I have numbness or tingling in my hands”) experienced in the past 7 days using a 5‐point Likert scale from 0 (not at all) to 4 (very much), with higher individual item scores reflecting more severe symptoms. The total score was derived following standard procedures for scoring FACT instruments such that lower total scores reflect higher neurotoxicity. The total score was prorated for patients missing less than 50% of items; patients missing 50% or more items were coded as missing. A total score of 30 or less was considered to indicate concerning neurotoxicity^15^, ^16^; 25 or less was considered clinically significant.^17^ Internal consistency in this sample was good (Cronbach's *α* = 0.77‐0.78).

#### 2.3.2 Adherence

Patients received up to 4 cycles of therapy (1 cycle = 3 weeks). Veliparib vs placebo was prescribed at 100 mg by mouth twice a day for the first 7 days of a cycle. Therefore, the total potential dose of oral therapy (veliparib or placebo) was 5600 mg (1400 mg/cycle*4 cycles). In this report, adherence was defined as receiving at least 90% of the full dose during the whole treatment period (ie receiving at least 5040 mg of veliparib or placebo), which was assessed using study treatment forms (ie documentation of dosage and administration of treatment). The proportion of adherence was calculated as number of patients with adherence divided by all treated patients. Patients who received less than 90% of the planned 5600 mg due to adverse events, withdrawal, or dose reductions were considered in the denominator, as these are indicators of treatment tolerability.

#### 2.3.3 CTCAE rated neuropathy

Patients with a CTCAEv.4.0 grade ≥ 1 on neurotoxicity items (peripheral sensory neuropathy, peripheral motor neuropathy) were classified as having neuropathy. Patients with CTCAE grade = 0 were classified as not having neuropathy. For sensitivity analysis, treatment‐related neurotoxicity was defined as neurotoxicity with an attribution code of possibly, probably or definitely.

### 2.4 Analyses

All analyses were conducted in the eligible and treated patient population. A two‐sample t‐test was used to compare the change in total FACT/GOG‐Ntx scores between baseline and follow‐up visits between the two treatment arms. At each time point, FACT/GOG‐Ntx total scores were compared between the treatment arms using Wilcoxon rank sum tests. A multivariable linear mixed effects model with an unstructured covariance matrix and random intercept was used to estimate the average difference in FACT/GOG‐Ntx total scores between the two arms, assuming that any missing data were missing at random. Time was dummy coded and a treatment‐by time interaction was included to test whether differences between treatment arms depended on time. As a sensitivity analysis, we also implemented a joint multivariate random‐effects model analysis to account for potentially different distributions of missing data between the two treatment arms.^18^

Individual FACT/GOG‐Ntx items were examined to explore treatment emergent symptoms of neurotoxicity. The proportion of patients who reported no symptom at baseline (item response = 0 [not at all]) and then rated the symptom as present (>1 [“a little bit”] or more) at 3‐ and 6‐months was calculated for each item to identify emerging symptoms. Only patients with neurotoxicity data at both baseline and follow‐up visits were included in these analyses. In addition, FACT/GOG‐Ntx individual items were also examined to identify the most common moderate or severe symptoms (ie FACT/GOG‐Ntx item response = 3 [quite a bit] or 4 [very much]) at 3‐ and 6 months. Treatment arm comparisons were conducted using Fisher exact test for these individual item analyses. Trend analysis about the proportion of moderate or severe symptoms was explored via a univariate logistic regression model with time being coded as 1(baseline), 2 (3 months), and 3 (6 months) and included as a continuous variable.

The proportion of adherent patients was calculated for each arm and compared between arms using Fisher exact test.

No adjustment was made for multiple comparisons. All significance tests were two‐sided with a type I error of 5%. Analyses were conducted with STATA 15.0.

Sample size was based on the trial's primary outcome analysis for progression free survival.^3^

Individual participant data may be made available upon request as per the ECOG‐ACRIN Data Sharing Policy.

## 3 RESULTS

### 3.1 Study population

Patient and disease characteristics were similar between the two treatment arms at baseline (see Owonikoko et al^3^). Of the 147 patients enrolled to the trial, 128 were eligible and received at least one dose of the treatment and served as the primary analysis population for this PRO study. The 19 patients excluded from this study's analysis were similar to the analyzed sample of 128 patients with respect to patient and disease characteristics (Table 1). However, they had significantly worse survival outcomes than the analyzed sample (Median PFS = 2.8 months, 95% CI = 0.5, 5.7 vs 5.9 months, 95% CI = 5.5, 6.1; Median OS = 4.8 months, 95% CI = 0.8, 6.5 vs 9.8 months, 95% CI = 8.8, 11.1). Patients in the analyzed sample were a median of 66 years of age and predominantly white (92.2%), and the majority (71%) had an ECOG PS of 1 at baseline. Male and female participation was similar (51.6% and 48.4%, respectively).

**TABLE 1:** Patient and disease characteristics in primary analysis sample and excluded patients

Variable	128 analyzable patients		19 patients excluded		*P* value
	N	%	N	%	
Age (y), median(range)	66 (45‐88)		64 (43‐79)		.42
Sex					.73
Female	62	48.4	10	52.6	
Male	66	51.6	9	47.4	
Race					.54
White	118	92.2	17	89.5	
Other	10	7.8	2	10.5	
Ethnicity					.70
Non‐Hispanic	124	96.9	18	94.7	
Other	4	3.1	1	5.3	
					
ECOG PS					.24
0	37	28.9	8	42.1	
1	91	71.1	11	57.9	
Weight loss in last 6 mo					.72
<5% of body weight	90	70.3	12	63.2	
5 to <10% of body weight	23	18.0	4	21.1	
10 to <20% of body weight	12	9.4	3	15.8	
≥20% of body weight	3	2.3	0	0.0	
LDH					.33
Normal	41	32.0	4	21.1	
Abnormal	87	68.0	15	79.0	
Pleural Effusion					.65
Absent	81	63.3	11	57.9	
Present	47	36.7	8	42.1	
Clinical outcome					
PFS, median (95%CI)	5.9 (5.5,6.1)		2.8(0.5,5.7)		.01
OS, median (95% CI)	9.8(8.8,11.1)		4.8(0.8,6.5)		.002

### 3.2 Pro completion rates

**FIGURE 1:**
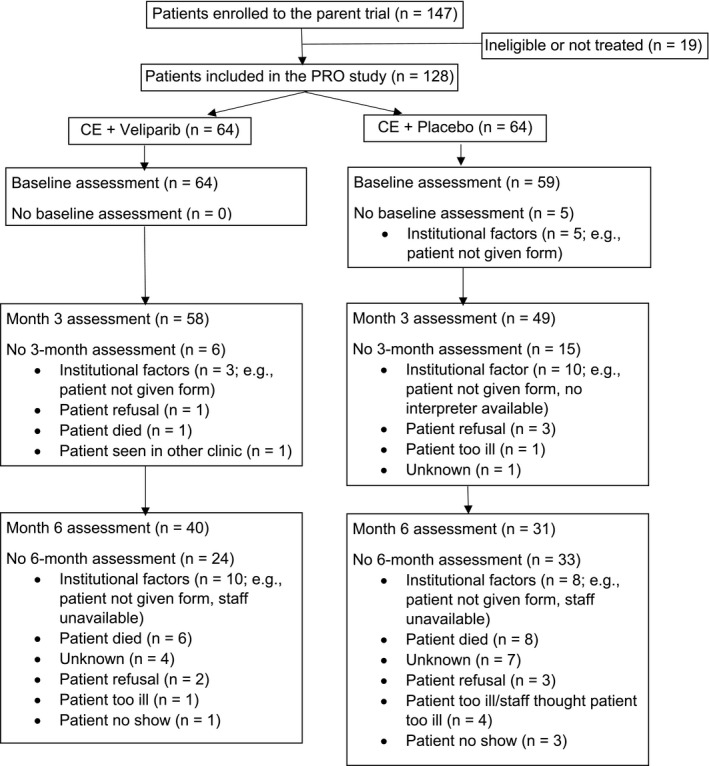
Consolidated Standards of Reporting Trials (CONSORT) Flowchart. PRO = Patient‐reported outcome. CONSORT flow diagram depicting study recruitment and retention. See text for patient‐reported outcome completion rates calculated with a denominator of those alive at each time point

Figure 1 (CONSORT) shows the number of assessments completed at each time point and reasons for non‐completion.

To calculate PRO completion rates, all patients who were alive at the scheduled assessment were expected to complete the FACT/GOG‐Ntx and used as the denominator. The PRO completion rate was 96% (123/128) at baseline, 84% (107/127) at 3 months, and 62% (71/114) at 6 months. Non‐completion was mainly attributable to institutional factors (eg patient not given form; n = 36; 57%), though patient refusal (n = 9; 13%) and patient illness (n = 6; 9%) also occurred. Across all time points, only one patient did not answer all 11 items of the FACT/GOG‐Ntx; this patient's score was prorated for the 6‐month assessment. Compliance with PRO completion was similar between the two arms at baseline (CE + V = 64; CE + *P* = 59), 3‐month (CE + V = 58; CE + *P* = 49), and 6‐month assessments (CE + V = 40; CE + *P* = 31).

### 3.3 Primary objective

#### 3.3.1 Comparison of patient‐reported neurotoxicity (FACT/GOG‐Ntx) by treatment arm

**FIGURE 2:**
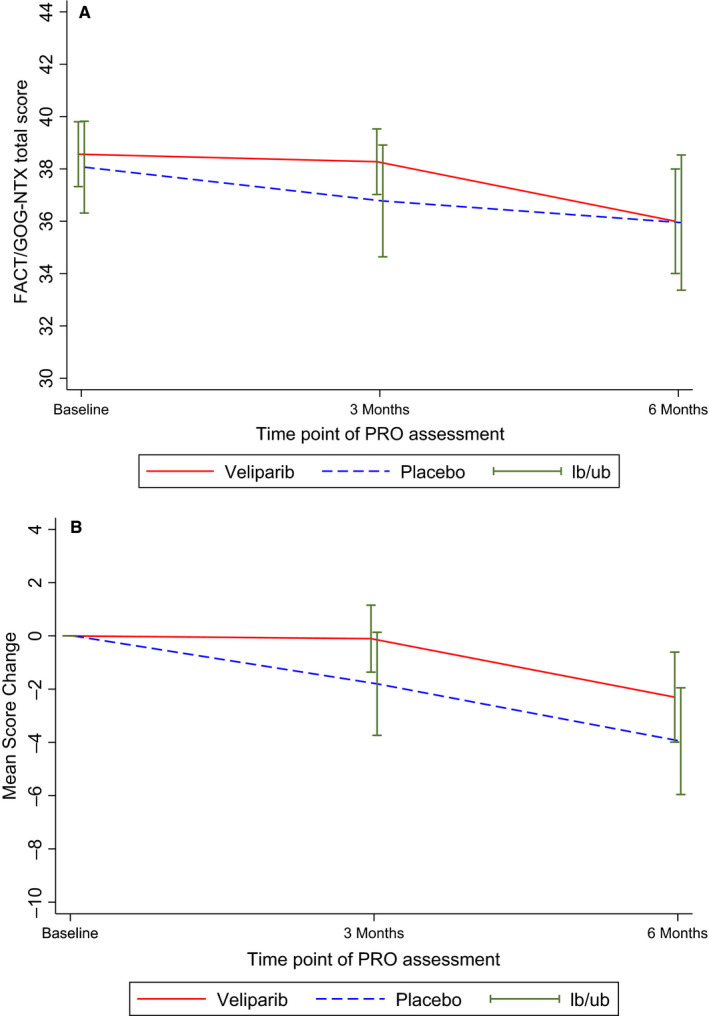
Mean and 95% CI of FACT/GOG‐Ntx neurotoxicity total score and total change score from baseline by treatment arm in eligible and treated patients. FACT/GOG‐Ntx = Functional Assessment of Cancer Therapy/Gynecologic Oncology Group Neurotoxicity (11‐items). PRO = Patient‐reported outcome. LB/UB = lower bound/upper bound of 95% confidence interval. Lower scores on the FACT/GOG‐Ntx indicate higher neurotoxicity. No statistically significant differences were observed between treatment arms in mean total neurotoxicity scores or in mean neurotoxicity change scores. The placebo arm experienced worsening neurotoxicity between baseline and 3‐month assessment

Patients reported similar neurotoxicity levels on the FACT/GOG‐Ntx at baseline in the two arms (CE + V = 38.6, SD = 5; CE + *P* = 38.1, SD = 6.7) with a mean score of 38.3 (SD = 5.9) for the overall sample. As shown in Table 2 and Figure 2A, there was no statistically significant difference in total neurotoxicity between arms at either 3‐month (CE + V = 38.3, SD = 4.8; CE + *P* = 36.8, SD = 7.4) or 6‐month assessment (CE + V = 36.0, SD = 6.2; CE + *P* = 35.9, SD = 7.0; *P*'s > .05), though the placebo arm reported worsening toxicity (ie lower scores on the FACT/GOG‐Ntx) from baseline to 3 months. There were also no statistically significant differences observed between the arms in change scores at either 3‐month (*P* = .13) or 6‐month (*P* = .20) assessments (Table 2, Figure 2B).

**TABLE 2:** Descriptive statistics for FACT/GOG‐Ntx neurotoxicity total scores and change scores in eligible and treated patients

Total scores at each assessment	Veliparib				Placebo				*P‐*value for between arm comparisons		
	N	Mean	SD	Median	n	Mean	SD	Median			
Baseline	64	38.6	5.0	39.5	59	38.1	6.7	40	.70		
Month 3	58	38.3	4.8	39.5	49	36.8	7.4	39	.57		
Month 6	40	36.0	6.2	37.5	31	35.9	7.0	37	.87		

Change scores	N	Mean change	SD		n	Mean change	SD		Differences in mean changes (veliparib—placebo)	SE	*P*‐value
Month 3 ‐ baseline	58	−0.1	4.8		45	−1.8	6.4		1.7	1.1	.13
Month 6‐baseline	40	−2.3	5.3		30	−4.0	5.4		1.6	1.3	.20

Sensitivity analysis results from the linear mixed‐effect model were largely the same (Table 3). Whereas the placebo group experienced worsening neurotoxicity (ie lower scores on the FACT/GOG‐Ntx) from baseline to 3 months (difference = −1.5, *P* = .045) and baseline to 6 months (difference = −3.1, *P* = .001), the veliparib group reported stable neurotoxicity from baseline to 3 months (difference = −0.2, *P* = .778) and worsening neurotoxicity from baseline to 6 months (difference = −2.4, *P* = .0003). There was no statistically significant difference in the total score between arms at either month 3 (difference = 2.1, *P* = .072) or month 6 (difference = 1.4, *P* = .332) after adjusting for baseline patient and disease characteristics. Marginally significant covariates included baseline ECOG performance status and race/ethnicity. Patients with worse performance status at baseline (ECOG 1 vs 0) reported higher toxicity (difference = −1.97, *P* = .07) as did non‐Hispanic White patients (difference = −2.72, *P* = .106). Results from the joint model analysis were similar to the linear mixed effect model (not shown).

**TABLE 3:** Linear mixed effect model analysis of FACT/GOG‐Ntx neurotoxicity total score

Covariates	Coef.	95% CI		*P* value
Treatment (veliparib vs placebo)	0.73	−1.26	2.72	.474
Time				
3 mo vs baseline	−1.54	−3.04	−0.04	.045
6 mo vs baseline	−3.07	−4.88	−1.25	.001
Treatment‐by‐time interaction				
3 mo & veliparib	1.34	−0.68	3.37	.194
6 mo & veliparib	0.62	−1.80	3.04	.614
Age (years, continuous)	0.03	−0.07	0.13	.582
Sex (male vs female)	−0.57	−2.45	1.31	.553
Race/ethnicity (non‐Hispanic White vs other)	−2.72	−6.03	0.58	.106
ECOG PS (1 vs 0)	−1.97	−4.09	0.16	.070
LDH (abnormal vs normal)	−0.11	−2.09	1.87	.916
Weight loss in previous six months (yes vs no)	−0.73	−2.72	1.26	.471
Pleural Effusion (present vs absent)	−0.74	−2.72	1.24	.464

Note

For dichotomous variables, the second group listed is the reference group (eg placebo is the reference group for treatment). Positive coefficient indicated more neurotoxicity in the reference category for a covariate, and negative coefficient indicated less neurotoxicity in the reference category.

### 3.4 Secondary objectives

#### 3.4.1 Treatment‐emergent Patient‐reported Symptoms (FACT/GOG‐Ntx)

**FIGURE 3:**
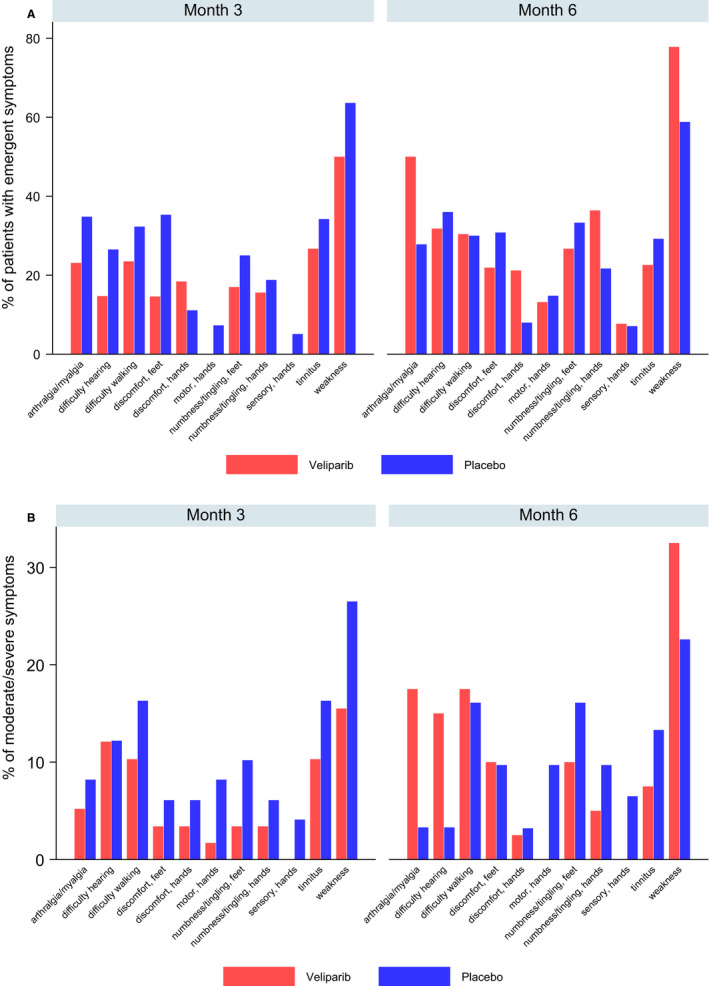
A Treatment‐emergent patient‐reported neurotoxicity symptom at 3 and 6 mo based on individual FACT‐GOG‐NTX items (% of patients rating as ≥“a little bit”) by treatment arm. FACT/GOG‐Ntx = Functional Assessment of Cancer Therapy/Gynecologic Oncology Group Neurotoxicity (11‐items). 3‐month assessment corresponds to end of cycle 4. 6‐mo assessment corresponds to 3‐mo post‐treatment. Items from the FACT/GOG‐NTX are as follows: difficulty walking = AN6; weakness = HI12; numbness/tingling, hands = NTX1; numbness/tingling, feet = NTX2; discomfort, hands = NTX3; discomfort, feet = NTX4; arthralgia/myalgia = NTX5; difficulty hearing = NTX6; tinnitus = NTX7; motor, hands = NTX8; sensory, hands = NTX9. Weakness was the most common treatment‐emergent symptom at 3 mo (50.0% in veliparib; 63.6% in placebo) and 6 mo (77.8% in veliparib; 58.8% in placebo). B, Most common moderate or severe patient‐reported neurotoxicity symptoms at 3 and 6 mo based on individual FACT‐GOG‐NTX items (% of patients rating as “quite a bit” or “very much”) by treatment arm. FACT/GOG‐Ntx = Functional Assessment of Cancer Therapy/Gynecologic Oncology Group Neurotoxicity (11‐items). 3‐month assessment corresponds to end of cycle 4. 6‐month assessment corresponds to 3‐months post‐treatment. Items from the FACT/GOG‐NTX are as follows: difficulty walking = AN6; weakness = HI12; numbness/tingling, hands = NTX1; numbness/tingling, feet = NTX2; discomfort, hands = NTX3; discomfort, feet = NTX4; arthralgia/myalgia = NTX5; difficulty hearing = NTX6; tinnitus = NTX7; motor, hands = NTX8; sensory, hands = NTX9. The proportion of patients endorsing moderate or severe symptoms (ie item response = 3 [quite a bit] or 4 [very much] did not differ significantly between treatment arms at any time point. Weakness was most common moderate or severe symptom reported at 3‐months (15.5% in veliparib; 26.5% in placebo) and 6‐month (32.5% in veliparib; 22.6% in placebo)

See Figure 3A for the proportion of patients experiencing treatment‐emergent symptoms in each arm at 3‐ and 6‐month assessments. At 3 months (cycle 4), the proportion of patients who reported treatment‐emergent symptoms (ie FACT/GOG‐Ntx = 0 [“not at all”] at baseline and ≥ 1 [“a little bit” or more] at 3 months) was highest for weakness (50.0%, 63.6%), discomfort in feet (14.6%, 35.3%), joint pain or muscle cramps (23.1%, 34.8%), ringing or buzzing in the ears (26.7%, 34.2%), and difficulty walking (23.5%, 32.3%) in the veliparib and placebo arms, respectively. Except for hand discomfort (18.4% veliparib, 11.1% placebo), a higher proportion of patients in the placebo arm experienced treatment‐emergent symptoms for all symptoms, though only foot discomfort (14.6% veliparib, 35.3% placebo, *P* = .036) was statistically different between arms.

At 6 months, the proportion of patients who reported treatment‐emergent symptoms was highest for weakness (77.8% veliparib, 58.8% placebo). With the exception of difficulty walking (30.4% veliparib, 30.0% placebo), the prevalence of other treatment‐emergent symptoms, while not statistically different between arms, were not consistently distributed between arms. After weakness, the next most prevalent treatment‐emergent symptoms were joint pain or muscle cramps (50.0%) and numbness or tingling in hands (36.4%) for patients on veliparib, and they were difficulty hearing (36.0%) and numbness or tingling feet (33.3%) for patients on placebo.

#### 3.4.2 Moderate or Severe Symptoms (FACT/GOG‐Ntx) at 3, 6 months

The proportion of patients endorsing moderate or severe symptoms on FACT/GOG‐Ntx items (ie item response = 3 [quite a bit] or 4 [very much]) did not differ significantly between treatment arms at any time point.

See Figure 3B for the proportion of patients experiencing moderate or severe symptoms in each arm at 3‐ and 6‐month assessments. Among the 11 items assessing neurotoxicity, the proportion of patients reporting moderate or severe symptoms at 3 months was highest for weakness (15.5%, 26.5%), difficulty walking (10.3%, 16.3%), trouble hearing (12.1%, 12.2%), and ringing or buzzing in the ears (10.3%, 16.3%) for veliparib and placebo, respectively. At 6 months, the proportion was highest for weakness (32.5%, 22.6%), difficulty walking (17.5%, 16.1%), joint pain and muscle cramps (17.5%, 3.3%), numbing or tingling in feet (10.0%, 16.1%), trouble hearing (15.0%, 3.3%), and ringing or buzzing in the ears (7.5%, 13.3%) for veliparib and placebo, respectively. In the placebo arm, the proportion of moderate or severe symptoms significantly increased over time for numbing or tingling in the hands (*P* = .029) and significantly decreased over time for joint pain and muscle cramps (*P* = .008).

#### 3.4.3 Adherence to oral therapy

Of the 128 eligible and treated patients, 103 (80%) received four cycles of treatment per protocol (53 on veliparib, 50 on placebo). Patients who did not receive four cycles due to death (n = 4), progression (n = 6), alternative therapy (n = 1), or complicating disease (n = 1) were excluded from the denominator of potentially adherent patients, as 4 cycles were not medically indicated and therefore these patients could not receive at least 90% of the planned dose. Patients who did not receive four cycles due to AEs (n = 9) or patient withdrawal (n = 4; reason unknown) were considered in the denominator of potentially adherent patients as AEs and patient withdrawal are indicators of tolerability. Therefore, there were 116 patients (59 veliparib; 57 placebo) who were eligible for four cycles of treatment and could have been adherent. Of these 116, 87 (75%; 48 on veliparib, 39 on placebo) were adherent (ie received at least 90% of the full dose). There was no statistically significant difference in adherence between arms (81% veliparib vs 68% placebo, *P* = .135).

#### 3.4.4 Comparison of CTCAE neuropathy and patient‐reported neurotoxicity

For CTCAE neuropathy, patients with a CTCAE grade ≥ 1 on any neurotoxicity items in CTCAE v4.0 (e.g, peripheral sensory neuropathy, peripheral motor neuropathy) were classified as having neuropathy. CTCAE grade 0 was classified as not having neuropathy. Of the 132 treated patients, 33 (25%; 20 on veliparib, 13 on placebo) experienced neuropathy per CTCAE assessments while on treatment or during follow‐up. There was no grade 3 or higher neuropathy reported; 77 episodes (64 peripheral sensory; 39 on veliparib, 25 on placebo; 13 peripheral motor; 8 on veliparib, 5 on placebo) of grade 1‐2 neuropathy were reported for these 33 patients.

Comparing FACT/GOG‐Ntx neurotoxicity scores reported at 3‐months (ie end of cycle 4) by CTCAE neuropathy reported in cycle 4, the mean FACT/GOG‐Ntx score was 35.8 (SD = 6.7) among 15 patients with grade 1 or 2 neuropathy per CTCAE in cycle 4, compared to a mean score of 37.9 (SD = 6.0) among the 95 patients without grade 1 or 2 neuropathy per CTCAE in cycle 4 (*P *> .05). Among the patients without neuropathy per CTCAE in cycle 4, 11 (12%) reported a FACT/GOG‐Ntx neurotoxicity score of 30 or less (concerning for neurotoxicity), and 3 (3%) reported a neurotoxicity score of 25 or less (clinically significant neurotoxicity).

Among patients with grade 1‐2 neuropathy per CTCAE in cycle 4, the most common moderate to severe (ie FACT/GOG‐Ntx item response = 3 [quite a bit] or 4 [very much]) symptoms patients reported on the FACT/GOG‐Ntx were weakness (40.0%), numbness in feet (20%), difficulty walking (13.4%), ringing or buzzing in the ears (13.4%), joint pain or muscle cramps (13.3%), and feet discomfort (13.3%). Among patients without grade 1‐2 neuropathy per CTCAE in cycle 4, the most common moderate to severe symptoms patients reported on the FACT/GOG‐Ntx were weakness (16.9%), ringing or buzzing in the ears (13.7%), difficulty walking (12.6%), and trouble hearing (12.6%). Results were similar when only treatment‐related neuropathy (ie toxicity probably, likely, or definitely attributed to treatment) on the CTCAE was considered (results not shown).

## 4 DISCUSSION

The ECOG‐ACRIN Cancer Research Group recently reported that the addition of veliparib to CE may have a role in the treatment of a subgroup of patients with treatment naïve ES‐SCLC, based on superior outcomes and a toxicity profile that is generally comparable to standard therapy based on physician‐rated adverse events.^3^ The goal of this study was to compare neurotoxicity between treatment arms using a patient‐reported outcome measure for a precise and robust measure of neurotoxicity to test the trial's hypothesis that veliparib would have a neuroprotective effect compared to placebo. Treatment arm comparisons were conducted at 3 months (end of cycle 4) and 6 months (3 months post‐treatment). Important secondary objectives were to identify treatment‐emergent and the most prevalent moderate to severe symptoms of neurotoxicity, compare adherence to oral therapy between arms, and compare CTCAE rated neurotoxicity to patient‐reported neurotoxicity.

Patient‐reported neurotoxicity was comparable between treatment arms based on similar mean levels of neurotoxicity at 3 and 6 months, magnitude of change in neurotoxicity symptoms from baseline to 3 and 6 months, and proportion of patients endorsing moderate to severe neurotoxicity at 3 and 6 months. Thus, the hypothesis that the addition of veliparib to CE would have a neuroprotective effect was not supported. However, whereas neurotoxicity scores worsened in the placebo arm between baseline and 3 months, they appeared stable in the veliparib arm during this time. This suggests a signal for a neuroprotective effect while patients were taking veliparib. This might explain why neurotoxicity worsened in the veliparib arm at 6‐months, as by that time patients had been off veliparib for 3 months. Data from larger ongoing trials (eg ClinicalTrials.gov NCT02289690) and correlative studies of SNPs that have been associated with increased risk for chemotherapy‐induced neurotoxicity (eg SOX10^19^) may help elucidate veliparib's potential neuroprotective effects. However, future studies seeking to evaluate veliparib's potential neuroprotective effects should include measures of patient‐reported toxicity. In this trial, 3% of patients categorized as not having neuropathy based on CTCAE grade (0) had patient‐reported neurotoxicity scores in the clinically significant range; 12% had scores that were comparable to those found in samples with known chemotherapy‐induced neuropathy.^16^ This supports prior findings that CTCAE grading may underestimate symptom severity and affirms the value in using PROs to capture patients’ experiences with treatment.

This study adds to limited literature on patient‐reported experiences of neurotoxicity among ES‐SCLC patients. In this sample, patients with a worse ECOG performance status (1 vs 0) at baseline appeared to experience worse neurotoxicity, as did non‐Hispanic White patients. Future studies should evaluate neurotoxicity among those with PS 2, which represent over 30% of lung cancer patients presenting for treatment,^20^ and in regimens using carboplatin, which tends to have fewer neuropathy side effects compared to cisplatin. The relation between race and neurotoxicity also warrants further study, as others have found race‐based differences in neurotoxicity in breast cancer.^21^

This study yielded information about the onset, trajectory, and severity of treatment‐related symptoms. As we begin to combine chemotherapy with other novel agents, it will be important to understand how such regimens impact ability to remain on and complete therapy. Most patients in both arms reported new onset of treatment‐related weakness from baseline to 3 months. Other common treatment‐related symptoms that emerged from baseline to 3 months included foot discomfort, arthralgia and myalgia, tinnitus, and difficulty walking, which were more prevalent in the placebo arm, though not statistically different. Many patients also reported symptoms at 6 months, after treatment had ended. Most patients in both arms reported new onset of treatment‐related weakness from baseline to 6 months. Other common treatment‐emergent symptoms at 6 months included arthralgia and myalgia, numbness or tingling in the hands, difficulty hearing, numbness or tingling in the feet, and difficulty walking. With the exception of difficulty walking, these symptoms were inconsistently distributed between treatment arms. Of the symptoms patients experienced, difficulty walking and ototoxicity were common at moderate to severe levels at 3 months. Weakness was the most prevalent moderate to severe symptom at 3 months and 6 months. Consistent with the trial's findings,^3^ patient‐reported weakness was not significantly different between treatment arms in this study, which further supports veliparib's tolerability.

The final marker of tolerability examined was adherence to oral therapy. Adherence to oral therapy (ie veliparib or placebo) was good. Most patients received at least 90% of the full dose of oral therapy prescribed. Although often not examined in studies of ES‐SCLC patients for whom treatment and consequently long‐term survival is limited, oral agents are increasingly used in treating cancer. These agents offer increased convenience for patients, but are also subject to elective discontinuation if patients are unable to tolerate their side effects.^22^, ^23^ Continued effort to understand adherence to new treatment regimens may identify patients who need better toxicity management, and ultimately maximize their potential therapeutic benefit.

Of concern, comparison of patient‐reported to physician grade neurotoxicity (ie CTCAE) suggested that 3%‐12% of patients may experience concerning neurotoxicity that is not captured by physician assessment. Further, our results suggest between 12% and 17% of patients who physicians classify as not having neurotoxicity may experience moderate to severe symptoms. The proportion of patients reporting moderate or severe weakness in either treatment arm was higher than CTCAE‐rated neurotoxicity of any grade at cycle 4. In the placebo arm, the proportion reporting moderate or severe difficulty walking and tinnitus was also higher than CTCAE‐rated neurotoxicity of any grade at cycle 4. Examination of individual patient‐reported items indicated patients who physicians identify as having neurotoxicity report worse weakness and foot numbness. This may suggest these symptoms are more likely to be included in providers’ assessment and conceptualization of chemotherapy‐induced neuropathy^24^; however, from the patient perspective, other moderate to severe symptoms at the end of treatment also included difficulty walking and ototoxicity, which may be overlooked in older patient populations. However, ototoxicity is a significant concern, as it can impair patient quality of life and function^25^, ^26^ and increase risk of falls and cognitive decline.^25^, ^27^, ^28^, ^29^ Assessment of specific symptoms and their functional impact should be incorporated into discussions of patient priorities and treatment plans.^30^, ^31^

### 4.1 Limitations

Given the small sample size, this study was only powered to detect moderate to large effect sizes. PRO assessment completion rates were poor at 6 months, which may have introduced sample bias. However, non‐completion was mainly due to institutional factors (eg failure to give a patient the assessment to complete, staff unavailability) and PRO completion rates were comparable between arms. The potential signal for non‐Hispanic Whites to experience worse overall neurotoxicity is exploratory. Other limitations include single items to assess specific symptoms and lack of items to directly assess impact of treatment side effects. Future trials assessing neurotoxicity should consider items to assess symptom bother, which itself may predict treatment discontinuation.^32^

## 5 CONCLUSION

This study suggests the addition of veliparib to CE is tolerable according to patient‐reported neurotoxicity, CTCAE rated neurotoxicity, and adherence to oral therapy. Patient‐reported weakness should be monitored. Future studies of veliparib should include patient‐reported measures of neurotoxicity, including the NCI PRO‐CTCAE^TM^, and consider complementary objective measures of neurotoxicity^33^ to further assess its potential neuroprotective effects. Item‐level analysis of symptoms of neurotoxicity in future trials may help identify early indicators of neurotoxicity during treatment, which may aid clinical management efforts. Providers should be aware of the potential for underestimating other likely moderate to severe symptoms of neurotoxicity, including ototoxicity.

## DISCLAIME

The content is solely the responsibility of the authors and does not necessarily represent the official views of the National Institutes of Health. Individual participant data may be made available upon request as per the ECOG‐ACRIN Data Sharing Policy.

## CONFLICT OF INTEREST

Drs. McLouth and Zhao have no conflicts of interest to disclose. Dr Owonikoko discloses a consulting or advisory role with: Novartis, Celgene, Eli Lilly, Sandoz, Abbvie, Eisai, G1 Therapeutics, Takeda, Seattle Genetics, Bristol‐Myers Squibb, MedImmune. Institutional research funding with: Novartis, Astellas Pharma, Celegene, Bayer Stem CentRx, Regeneron, AstraZeneca/MedImmune, Abbvie, G1 Therapeutics, and Bristol‐Myers Squibb. Patients, Royalties, and Other Intellectual Property with: Overcoming Acquired Resistance to Chemotherapy Treatments Through Suppression of STAT3 (Institutional), Selective Chemotherapy Treatments and Diagnostics Methods Related Thereto (Institutional). Dr Feliciano discloses a consulting or advisory role with: Genentech, AstraZeneca, Merck, Takeda, and Pfizer. Dr Mohindra discloses a consulting or advisory role with: Genentech, Abbvie, and AstraZeneca. Dr Dahlberg discloses a grant from the EA statistical center grant that supported this work, consulting or advisory role to AstraZeneca, and a patent pending for a statistical model assessing tumor growth (Institutional). Dr Wade discloses immediate family member employment with Johnson & Johnson, and stock or other ownership interests with: Celgene, Abbott (immediate family member), GlaxoSmithKline (immediate family member), Johnson & Johnson (immediate family member), and Novartis (immediate family member). Dr Srkalovic discloses speakers’ bureau with Takeda and Janssen. Dr Leach discloses a consulting or advisory role with PRA International. Dr Leal discloses a consulting or advisory role with: Takeda, AstraZeneca, Novartis, Abbvie, Bristol‐Meyers Squibb, Bayer and Genentech and travel, accommodations, or expenses supported by Xcovery and Mirati Therapeutics. Dr Aggarwal discloses a consulting or advisory role with Genentech, Bristol‐Myers Squibb, Celgene, MedImmune, and institutional research funding supported by Incyte, Macrogenics, Merck Sharp & Dohme, and AstraZeneca/MedImmune. Dr Cella is President of FACIT.org and discloses research grants to his institution from Abbvie, Bristol‐Myers Squibb, PledPharma and Pfizer, as well as a consulting or advisory role with Abbvie, Pfizer, Novartis, PledPharma, and Asahi‐Kaseo. Dr Ramalingam discloses a consulting or advisory role with Amgen, Abbvie, Bristol‐Myers Squibb, Eli Lilly/ImClone, Genentech, Takeda, and Luxo, and research grants from AstraZeneca, Merck, and Tesaro. Dr Wagner discloses a consulting or advisory role with EveryFit, Janssen, and Celgene.

## AUTHOR CONTRIBUTIONS

Steffen McLouth, Zhao, Dahlberg, Owonikoko, Cella, Ramalingam, Wagner, Feliciano, Mohindra, Wade, Srkalovic, Lash, Leach, Leal, and Aggarwal contributed to conceptualization and methodology. Owonikoko, Ramalingam, and Wagner contributed to project administration and supervision. Zhao and Dahlberg contributed to data analysis. Steffen McLouth, Zhao, Dahlberg, Feliciano, Mohindra, and Wagner contributed to writing—original draft. Steffen McLouth, Zhao, Dahlberg, Owonikoko, Cella, Ramalingam, Wagner, Feliciano, Mohindra, Wade, Srkalovic, Lash, Leach, Leal, and Aggarwal contributed to writing—review and editing.

## Data Availability

Individual participant data may be made available upon request as per the ECOG‐ACRIN Data Sharing Policy.

## References

[1] Kalemkerian GP , Schneider BJ . Advances in small cell lung cancer. Hematol Oncol Clin North Am. 2017;31(1):143‐156. 27912830 10.1016/j.hoc.2016.08.005

[2] Bunn PA Jr , Minna JD , Augustyn A , et al. Small cell lung cancer: can recent advances in biology and molecular biology be translated into improved outcomes? J Thorac Oncol. 2016;11(4):453‐474. 26829312 10.1016/j.jtho.2016.01.012 PMC4836290

[3] Owonikoko TK , Dahlberg SE , Sica GL , et al. Randomized phase II trial of cisplatin and etoposide in combination with veliparib or placebo for extensive‐stage small‐cell lung cancer: ECOG‐ACRIN 2511 Study. J Clin Oncol. 2019;37(3):222‐229. 30523756 10.1200/JCO.18.00264 PMC6338394

[4] Atkinson TM , Rogak LJ , Heon N , et al. Exploring differences in adverse symptom event grading thresholds between clinicians and patients in the clinical trial setting. J Cancer Res Clin Oncol. 2017;143(4):735‐743. 28093637 10.1007/s00432-016-2335-9 PMC5354957

[5] Patrick DL , Burke LB , Powers JH , et al. Patient‐reported outcomes to support medical product labeling claims: FDA perspective. Value Health. 2007;10(Suppl 2):S125‐137. 17995471 10.1111/j.1524-4733.2007.00275.x

[6] Basch E . The missing voice of patients in drug‐safety reporting. N Engl J Med. 2010;362(10):865‐869. 20220181 10.1056/NEJMp0911494 PMC3031980

[7] Zatloukal P , Cardenal F , Szczesna A , et al. A multicenter international randomized phase III study comparing cisplatin in combination with irinotecan or etoposide in previously untreated small‐cell lung cancer patients with extensive disease. Ann Oncol. 2010;21(9):1810‐1816. 20231298 10.1093/annonc/mdq036

[8] Hong JS , Tian J , Wu LH . The influence of chemotherapy‐induced neurotoxicity on psychological distress and sleep disturbance in cancer patients. Curr Oncol. 2014;21(4):174‐180. 25089099 10.3747/co.21.1984 PMC4117615

[9] Kolb NA , Smith AG , Singleton JR , et al. The association of chemotherapy‐induced peripheral neuropathy symptoms and the risk of falling. JAMA Neurol. 2016;73(7):860‐866. 27183099 10.1001/jamaneurol.2016.0383 PMC6715416

[10] Hershman DL , Lacchetti C , Dworkin RH , et al. Prevention and management of chemotherapy‐induced peripheral neuropathy in survivors of adult cancers: American Society of Clinical Oncology clinical practice guideline. J Clin Oncol. 2014;32(18):1941‐1967. 24733808 10.1200/JCO.2013.54.0914

[11] Cavaletti G , Marmiroli P . Chemotherapy‐induced peripheral neurotoxicity. Nat Rev Neurol. 2010;6(12):657‐666. 21060341 10.1038/nrneurol.2010.160

[12] Brewer JR , Morrison G , Dolan ME , Fleming GF . Chemotherapy‐induced peripheral neuropathy: current status and progress. Gynecol Oncol. 2016;140(1):176‐183. 26556766 10.1016/j.ygyno.2015.11.011 PMC4698212

[13] Ilnytska O , Lyzogubov VV , Stevens MJ , et al. Poly(ADP‐Ribose) polymerase inhibition alleviates experimental diabetic sensory neuropathy. Diabetes. 2006;55(6):1686‐1694. 16731831 10.2337/db06-0067 PMC2228258

[14] Drel VR , Lupachyk S , Shevalye H , et al. New therapeutic and biomarker discovery for peripheral diabetic neuropathy: PARP inhibitor, nitrotyrosine, and tumor necrosis factor‐{alpha}. Endocrinology. 2010;151(6):2547‐2555. 20357221 10.1210/en.2009-1342 PMC2875829

[15] Calhoun EA , Welshman EE , Chang C‐H , et al. Psychometric evaluation of the Functional Assessment of Cancer Therapy/Gynecologic Oncology Group—Neurotoxicity (Fact/GOG‐Ntx) questionnaire for patients receiving systemic chemotherapy. Int J Gynecol Cancer. 2003;13(6):741‐748. 14675309 10.1111/j.1525-1438.2003.13603.x

[16] Cella D , Huang H , Homesley HD , et al. Patient‐reported peripheral neuropathy of doxorubicin and cisplatin with and without paclitaxel in the treatment of advanced endometrial cancer: results from GOG 184. Gynecol Oncol. 2010;119(3):538‐542. 20863554 10.1016/j.ygyno.2010.08.022

[17] Moore DH , Donnelly J , McGuire WP , et al. Limited access trial using amifostine for protection against cisplatin‐ and three‐hour paclitaxel‐induced neurotoxicity: a phase II study of the Gynecologic Oncology Group. J Clin Oncol. 2003;21(22):4207‐4213. 14615449 10.1200/JCO.2003.02.086

[18] Pantazis N , Touloumi G . Analyzing longitudinal data in the presence of informative dropout: the Jmre1 command. Stata J: Promoting Commun Statistics Stata. 2010;10(2):226‐251.

[19] McWhinney‐Glass S , Winham SJ , Hertz DL , et al. Cumulative genetic risk predicts platinum/taxane‐induced neurotoxicity. Clin Cancer Res. 2013;19(20):5769–5776. 23963862 10.1158/1078-0432.CCR-13-0774 PMC3798385

[20] Lilenbaum RC , Cashy J , Hensing TA , Young S , Cella D . Prevalence of poor performance status in lung cancer patients: implications for research. J Thorac Oncol. 2008;3(2):125‐129. 18303431 10.1097/JTO.0b013e3181622c17

[21] Schneider BP , Li L , Radovich M , et al. Genome‐wide association studies for taxane‐induced peripheral neuropathy in ECOG‐5103 and ECOG‐1199. Clin Cancer Res. 2015;21(22):5082‐5091. 26138065 10.1158/1078-0432.CCR-15-0586 PMC4717479

[22] Geynisman DM , Wickersham KE . Adherence to targeted oral anticancer medications. Discov Med. 2013;15(83):231‐241. 23636140 PMC6477693

[23] Henry NL , Azzouz F , Desta Z , et al. Predictors of aromatase inhibitor discontinuation as a result of treatment‐emergent symptoms in early‐stage breast cancer. J Clinc Oncol. 2012;30(9):936‐942. 10.1200/JCO.2011.38.0261 PMC3341106 22331951

[24] Kuroi K , Shimozuma K , Ohashi Y , et al. A questionnaire survey of physicians’ perspectives regarding the assessment of chemotherapy‐induced peripheral neuropathy in patients with breast cancer. Jpn J Cancer Res. 2008;38(11):748‐754. 10.1093/jjco/hyn100 18845520

[25] Landier W . Ototoxicity and cancer therapy. Cancer. 2016;122(11):1647‐1658. 26859792 10.1002/cncr.29779

[26] Ciorba A , Bianchini C , Pelucchi S , Pastore A . The impact of hearing loss on the quality of life of elderly adults. Clin Interv Aging. 2012;7:159‐163. 22791988 10.2147/CIA.S26059 PMC3393360

[27] Le Saux O , Falandry CJ . Toxicity of cancer therapies in older patients. Curr Oncol Rep. 2018;20(8):64. 29896642 10.1007/s11912-018-0705-y

[28] Lin FR , Ferrucci L . Hearing loss and falls among older adults in the United States. Arch Gen Intern Med. 2012;172(4):369‐371. 10.1001/archinternmed.2011.728 PMC3518403 22371929

[29] Lin FR , Yaffe K , Xia J , et al. Hearing loss and cognitive decline in older adults. JAMA Intern Med. 2013;173(4):293‐299. 23337978 10.1001/jamainternmed.2013.1868 PMC3869227

[30] Bakitas MA . Background noise: the experience of chemotherapy‐induced peripheral neuropathy. Nurs Res. 2007;56(5):323‐331. 17846553 10.1097/01.NNR.0000289503.22414.79

[31] Tariman JD , Berry DL , Cochrane B , Doorenbos A , Schepp KG . Physician, patient, and contextual factors affecting treatment decisions in older adults with cancer and models of decision making: a literature review. Oncol Nurs Forum. 2012;39(1):E70‐83. 22201670 10.1188/12.ONF.E70-E83 PMC3247918

[32] Wagner LI , Zhao F , Goss PE , et al. Patient‐reported predictors of early treatment discontinuation: treatment‐related symptoms and health‐related quality of life among postmenopausal women with primary breast cancer randomized to anastrozole or exemestane on NCIC Clinical Trials Group (CCTG) MA.27 (E1Z03). Breast Cancer Res Treat. 2018;169(3):537‐548. 29455298 10.1007/s10549-018-4713-2 PMC6092930

[33] Mazzeo A , Pace A , Pessino A , et al. The chemotherapy‐induced peripheral neuropathy outcome measures standardization study: from consensus to the first validity and reliability findings. Ann Oncol. 2012;24(2):454‐462. 22910842 10.1093/annonc/mds329 PMC3551481

